# Health TAPESTRY Ontario: protocol for a randomized controlled trial to test reproducibility and implementation

**DOI:** 10.1186/s13063-020-04600-y

**Published:** 2020-08-14

**Authors:** Dee Mangin, Larkin Lamarche, Doug Oliver, Sivan Bomze, Sayem Borhan, Tracy Browne, Tracey Carr, Julie Datta, Lisa Dolovich, Michelle Howard, Sarah Marentette-Brown, Cathy Risdon, Samina Talat, Jean-Eric Tarride, Lehana Thabane, Ruta Valaitis, David Price

**Affiliations:** 1 Department of Family Medicine, McMaster University, David Braley Health Sciences Centre, 100 Main Street West, 5th floor, Hamilton, ON L8P 1H6 Canada; 2 Department of Family Medicine, McMaster University, David Braley Health Sciences Centre, 100 Main Street West, 3rd floor, Hamilton, ON L8P 1H6 Canada; 3 Canadian Red Cross, 5700 Cancross Court, Mississauga, ON L5R 3E9 Canada; 4 Department of Family Medicine, and Department of Health Research Methods, Evidence and Impact McMaster University, 1280 Main Street West, Hamilton, ON L8S 4L8 Canada; 5 Canadian Red Cross, 1460 Fairburn Street, Sudbury, ON P3A 1N7 Canada; 6 Department of Family Medicine, McMaster University, David Braley Health Sciences Centre, 100 Main Street West, 6th floor, Hamilton, ON L8P 1H6 Canada; 7 Department of Health Research Methods, Evidence and Impact, McMaster University, Programs for Assessment of Technologies in Health and Center for Health Economics and Policy Analysis, CRL 227, 1280 Main Street West, Hamilton, ON L8S 4L8 Canada; 8 Department of Health Research Methods, Evidence and Impact, McMaster University, Programs for Assessment of Technologist in Health, 1280 Main Street West, Hamilton, ON L8S 4L8 Canada; 9 School of Nursing, McMaster University, Health Sciences Centre, 1280 Main Street West, Hamilton, ON L8S 4L8 Canada

**Keywords:** Primary health care, Randomized controlled trial, Integrated care, Interdisciplinary health care teams, Health care volunteers, Older adults, Implementation

## Abstract

**Background:**

Health TAPESTRY (Health Teams Advancing Patient Experience: STRengthening qualitY) aims to help people stay healthier for longer where they live by providing person-focused care through the integration of four key program components: (1) trained volunteers who visit clients in their homes, (2) an interprofessional primary health care team, (3) use of technology to collect and share information, and (4) improved connections to community health and social services. The initial randomized controlled trial of Health TAPESTRY found promising results in terms of health care use and patient outcomes, indicating a shift from reactive to preventive care. The trial was based on one clinical academic center, thus limiting generalizability. The study objectives are (1) to test reproducibility of the established effectiveness of Health TAPESTRY on physical activity and hospitalizations, (2) to test the feasibility of, and understand the contributing factors to, the implementation of Health TAPESTRY in six diverse communities across Ontario, Canada, and (3) to determine the value for money of implementing Health TAPESTRY.

**Methods:**

This planned study is a pragmatic parallel randomized controlled trial with a delayed intervention for control participants at 6 months. This trial will simultaneously assess effectiveness and implementation in a real-world setting (type II hybrid) in six diverse communities across Ontario. Participants 70 years of age and older will be randomized into the Health TAPESTRY intervention or the control group (usual care). Intervention clients will receive an individualized plan of care from an interprofessional care team. The plan will be based on a client’s goals and current health risks identified through volunteer visits. The study’s outcomes are mapped onto the RE-AIM framework, with levels of physical activity and number of hospitalizations as the co-primary outcomes. The main analysis will be a comparison at 6 months.

**Discussion:**

It is important to evaluate the effectiveness and implementation of Health TAPESTRY in multiple communities prior to scaling or widespread adoption.

**Trial registration:**

ClinicalTrials.gov NCT03397836. Registered on 12 January 2018

## Background

With improved living circumstances and health care, the world’s population of older adults is increasing and expected to double by 2050 [1]. Older adults are consistently high users of the health care system and often have complex health needs [2]. As a result, health care systems and associated services are experiencing increasing pressure to meet patient needs in an effective and efficient way. Barriers to delivery of healthcare include a lack of continuity of care and coordinated transitions between health and social care domains, barriers to accessing community services, and poor coordination in addressing social determinants of health [2–4]. Primary care is the central focal point of the health care system for most patients. Primary care providers practice person-focused care (not disease-oriented care) over time for all conditions except very uncommon ones and coordinate and integrate their patients’ care regardless of where care takes place. Primary health care is central to a health care system that can function well to address diverse population needs and improve patient outcomes and is associated with increased population longevity and reduced health care distribution inequities [5–9]. Regardless of what aspect of primary health care is investigated (e.g., access, utilization, enrollment, or availability), the effects of investing in primary care are consistent [10]. Health care systems with strong primary care systems are also associated with more efficient use of services including: lower hospitalization rates, lower health care system costs, and sustainable system development [1]. Research has identified that the key elements that support these positive effects are person-focused care [11, 12], relational continuity [13–15], comprehensiveness [16, 17], first contact care [16], and care coordination [10, 18–20].

The Health TAPESTRY (Health Teams Advancing Patient Experience: STRengthening qualitY) program is a complex person-focused intervention that is anchored in primary care. Health TAPESTRY was designed with some of the current health system barriers in mind and to align with best practices from primary health care research [21, 22] and the Starfield’s principles of primary care delivery [5, 7]. Health TAPESTRY works to help people stay healthier for longer in the places where they live—the basis of engaging patients in a meaningful way is through a conversation about health goals and what matters most to patients in their lives. In an initial randomized controlled trial (RCT) that assessed effectiveness and cost-effectiveness, we found null results for the pre-specified primary outcome of goal attainment, but statistically significant improvements in pre-specified secondary outcomes related to health and health services use [23]. Specifically, we found that more minutes of walking per week and less time spent sitting per week were reported in the Health TAPESTRY group compared to the control group [24, 25]. We also found statistically significant differences in health service use with reduced hospitalizations and increased primary health care visits (including both family physicians and other health care providers) [25], suggesting that Health TAPESTRY may be shifting care from a reactive to a proactive approach.

The Health TAPESTRY program incorporates four parts:

1. Trained volunteers who meet with clients (i.e., patients enrolled in Health TAPESTRY) in their homes to discuss clients’ health and life goals and unidentified health and health-related social needs
2. The use of technology for collecting and sharing information with the primary care team
3. An interprofessional primary health care team (who meets regularly as a “TAP-Huddle”) to create individualized plans of care based on the data gathered, which is integrated with the patient’s longitudinal primary care
4. Community engagement and connections to assist clients to meet their goals and address health risks and needs.

A limitation of the initial RCT was it was only conducted in one established, well-supported academic interprofessional team environment [24]. Since primary health care is diverse in regard to team composition, work flow, and funding models, we wish to test the reproducibility of the results found in the initial trial of Health TAPESTRY set among a broader range of primary care clinics and communities.

### Grounding frameworks

We used the RE-AIM framework (Reach, Effectiveness or Efficacy, Adoption, Implementation and Maintenance (RE-AIM.org) to develop the objectives, research questions, and outcome measures for this implementation study and Normalization Process Theory (NPT) will help us understand the processes of implementation [26, 27]. RE-AIM is a well-recognized framework that has been widely used for evaluating the implementation of health programs [28–30]. The RE-AIM framework can be used in the planning, development, implementation, and evaluation phases of programs. We will use NPT to complement the RE-AIM framework and facilitate an understanding of the implementation process of the Health TAPESTRY approach into routine practice in the different communities [26, 27]. NPT has been applied in primary care settings to study implementation [31].

### Objectives and hypotheses

There are three objectives in this study, which are:

1. To test reproducibility of the effectiveness of Health TAPESTRY on health service utilization and physical activity compared to usual care that we found in our initial study [24, 25]
2. To test the feasibility of, and understand the contributing factors to, implementation of the Health TAPESTRY program in six diverse communities across Ontario, Canada
3. To determine the value for money of implementing Health TAPESTRY

We hypothesize that results will be reproduced from the first trial evaluation such that Health TAPESTRY will result in a reduction in hospitalizations and an increase in physical activity levels, compared to usual care at 6 months, and will be cost-effective compared to usual care.

## Methods/design

### Trial design

**Fig. 1:**
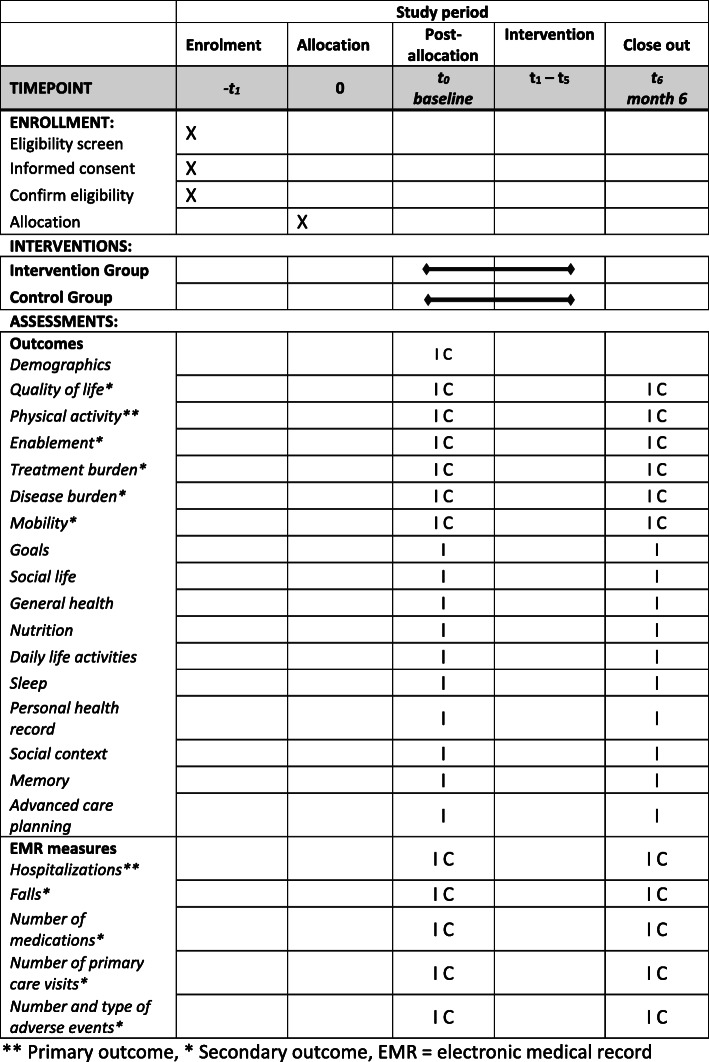
Study timeline and date collection time points

This study is a pragmatic parallel randomized controlled trial with a delayed intervention for control participants at 6 months. We will simultaneously assess effectiveness and implementation strategy in a real-world setting (type II hybrid) [32]. The main group comparisons will be at 6 months. We report this study protocol in accordance with the SPIRIT guidelines (see Additional file 1 for the checklist; see Fig. 1 for the SPIRIT Figure) [33] and TIDier checklist (see Additional file 2) [34]. We will use mixed-methods data collection, mapping onto the adoption, implementation, and maintenance domains in RE-AIM.

### Study setting

To be an implementation site, a primary care practice must meet a list of inclusion and exclusion criteria (Table 1). Included in the criteria is having partnership or access to an organization responsible for oversight of volunteers to implement the home visits and data collection in Health TAPESTRY.

**Table 1:** Health TAPESTRY implementation site inclusion and exclusion criteria

Primary care practice inclusion criteria	Primary care practice exclusion criteria
A primary care practice with a clearly identified practice champion for Health TAPESTRY model	No functional connections to an interdisciplinary primary care team to support individual patient assessments
Interprofessional primary care team available to provide core implementation components	Focused assessment on a single condition or disease
Team-based use of an electronic medical record system for documentation or willingness to engage in team-based use	
Able and willing to use the Health TAPESTRY web-based application (TAP-App)	
Partnership or access to a local organization with volunteer infrastructure with the capacity to recruit, train, sustain, and coordinate volunteers, and ensure volunteers have access to the digital health tools needed to fulfill role	

The study will be conducted within Family Health Teams (FHTs) in six communities across Ontario, Canada. A FHT is a type of primary care delivery model within Ontario that formally connects physicians and other health care professionals (e.g., dietitians, social workers, pharmacists) to improve the quality and effectiveness of primary health care services [35]. FHT team members are often co-located, but not always.

### Participant inclusion and exclusion criteria

Participants must be 70 years of age or older and rostered to a participating primary care physician. Participants will be excluded if they: live in a long-term care facility, are likely to be unavailable for follow-up for the 6-month study period, participated in the first implementation of Health TAPESTRY, or have other conditions or circumstances that would prevent them from engaging with the volunteers or completing the surveys with volunteers (e.g., severe dementia, inability to comprehend English even with caregiver translation). The inclusion and exclusion criteria for participants replicate those of the initial trial of Health TAPESTRY [24, 25].

### Recruitment

The main recruitment strategy is an EMR query run to identify all patients 70 years of age or older and rostered to physicians participating in Health TAPESTRY. Implementation sites (i.e., a participating primary care practice) will develop a recruitment strategy to identify eligible patients that fits the local context. Eligible patients will be mailed an information letter and consent form on behalf of participating physicians at each FHT inviting them to participate. Patients that return a signed consent form (a copy of the consent form is available from the corresponding author by request) will be contacted by a volunteer coordinator to confirm eligibility and review all information within the consent. Secondary recruitment strategies may include providing information in waiting rooms and printed materials for health care providers to hand out.

### Randomization and blinding

After informed consent is obtained, participants will be randomized by a volunteer coordinator into the intervention or control group with a ratio of 1:1 using blocked randomization with variable block sizes of 2, 4, and 6 through REDCap (Version 9.3.1, Vanderbilt University) [36]. Couples who are eligible and both decide to participate in the program will be allocated to the same group by randomizing one person and allocating the second person to the same group. For the main analysis, one person’s data will be randomly selected for inclusion. The health care providers will not be blinded; however, they will only know a participant is receiving the intervention once a Health TAPESTRY report is received. The people (volunteers, researchers) involved in research outcome data collection will not be blinded.

### Intervention

#### Procedures and data collection

A participant (termed “client” within the program), regardless of allocation, will receive a visit in their home from two volunteers at the start of the study and again 6 months later. The volunteers will collect data using structured surveys on the Health TAPESTRY web-based application (TAP-App) covering a variety of domains (i.e., physical activity, nutrition, mobility, quality of life; see Table 2). The volunteers will enter all data into the TAP-App using a tablet computer. The volunteers may return to the client’s home multiple times until all the surveys are complete. Participants may withdraw from the study at any point by informing the volunteer coordinator.

**Table 2:** List of all TAP-App surveys

Construct *Survey name*	Survey description	Key information for TAP-Report	Intervention		Control	
			T_**0**_	T_**6**_	T_**0**_	T_**6**_
Demographic information *Custom*	Basic demographics	NA	X		X	
Quality of life* *EQ 5D-5L* [37]	Quality of life with respect to mobility, self-care, usual activities, pain, and anxiety/depression. Five answer options provided from able to/none to extremely difficult/extreme levels (scores ranges 0–1, higher scores = higher quality of life)	• Severe problems in walking about or unable to walk about • Severe problems washing or dressing self or unable to wash or dress self • Severe problems doing usual activities or unable to do usual activities • Severe pain/discomfort or extreme pain/discomfort • Severely anxious/depressed or extremely anxious/depressed	X	X	X	X
Physical activity *International Physical Activity Questionnaire (IPAQ)* *(short)* [38]*	Time spent doing physical activity per week	Calculated time in moderate and vigorous physical activity Time spent sitting in one typical day (in hours)	X	X	X	X
*Rapid Assessment of Physical Activity (RAPA)* [39]	Quick assessment of physical activity for older adults (score range 1–7, higher scores = higher physical activity)	Suboptimal physical activity (score < 6)	X	X	X	X
Enablement* *Patient Enablement Instrument (PEI)* [40]	Client’s ability to cope with, or have Control over their health after visit with health care team	NA	X	X	X	X
Treatment burden* *Multimorbidity Treatment Burden Questionnaire (MTBQ)* [41]	Level of difficulty of 10 treatment tasks (e.g., taking many medications)	NA	X	X	X	X
Disease burden* *Disease Burden Morbidity Assessment* (*DBMA*)[42]	Level of limitation chronic diseases have on client’s daily activities	NA	X	X	X	X
Daily life activities *Custom*	Description of client’s daily activities, any need of assistance and general thoughts about current activities	Full text responses included	X			X
Goals *Custom*	General life or health based goals	Goals listed	X	X^+^		X
Social life *Friendship Scale/Custom* [43]*	Client’s relationships with others Added questions about social life (score range 0–24, lower score = higher risk)	• Social isolation risk score (score < 15) • Felt isolated from other people most of the time (or almost always) • Felt alone and friendless most of the time (or almost always) • Transportation challenges • Loss of a partner • Living alone • Finding it hard to make ends meet	X	X		X
General health *Edmonton Frail Scale* [44]*	Falls, need of assistance with self-care and household activities, mood, medications, weight, incontinence, fall risk (score range 0–15, higher score = more frail)	• Edmonton Frail Scale score indicated high risk (score 4-15) • Uses 5+ prescription medications • Often feels sad or depressed • Sometimes loses control of bladder • More than 20 s on timed up-and-go • Requires assistance for timed up-and-go • Has fallen in last year	X	X		X
Nutrition *Screen II (8-item)* [45]	Indicates if client has a nutritional problem or at risk of developing one (score range 0–64, lower score = higher nutritional risk)	• High nutritional risk score (score < 38) • Does not know own weight or if weight changed OR lost more than 10 pounds in the past 6 months OR gained more than 10 pounds in the past 6 months • Skips meals almost every day • Poor appetite • Sometimes/often/always coughs, chokes, or has pain when swallowing food or fluids	X	X		X
Mobility [46]	Level of mobility limitations	• Preclinical or minor or major limitation in walking 0.5 km • Preclinical or minor or major limitation in walking 2.0 km • Preclinical or minor or major limitation in climbing stairs	X	X		X
Sleep *15-D* (sleep item) [47]	Sleeping difficulties	• Great problems with sleep • Severe problems with sleep	X	X		X
Personal health record *Custom*	Interest in creating a personal health record	NA	X	X^+^		X
Social context *Custom)*	Description of client’s context	Descriptive response included	X			X
Memory *Custom*	Memory difficulties	Problems with memory impact daily activities	X			X
Advance care planning *Custom*	Client’s interest in discussing advance care planning with physician	Interested in having a discussion with family physician about advance care planning	X			X
Oral health¶ *Recommended Oral Health Screening Questions* [48]	Oral health	• Problems or pain with dentures • Oral hygiene risk • Has diet risk factor for poor oral health • Family history of tooth decay • Acid reflux • Oral dryness • Symptoms of active dental disease	X		X	
Smoking and alcohol^¥^ *Custom*	Smoking and alcohol behaviors	• Wants help to address smoking behavior OR wants help to address smoking behavior in the future OR does not want help to address smoking behavior • Wants help to address drinking behavior OR wants help to address drinking behavior in the future OR does not want help to address drinking behavior	X			
Health TAPESTRY experience* *Custom*	Feedback on, and impact of Health TAPESTRY program including negative effects	NA		X		
Community program and service use *Community Programs and Services* (adapted) [49]	Community program and services connected to through Health TAPESTRY	NA		X		

*NA* not applicable

*Survey is used as a research outcome measure

¶Survey only used at Hamilton FHT site

^¥^Survey only used at Dufferin Area FHT site

^+^Follow-up survey to previous responses

#### Intervention group

For the intervention group, once all baseline surveys are completed, the TAP-App will create a summary TAP-Report from the information provided. This summary includes the client’s reported goals, key information associated with their answers and/or specific survey scores (Table 2), and volunteer observations. The TAP-Report will be reviewed by a volunteer coordinator and then securely sent using the TAP-App to the TAP-Huddle at the client’s clinic. The TAP-Huddle is an interprofessional team of health care providers who meet approximately weekly. Each TAP-Huddle is responsible for reviewing the report and creating and implementing a plan of care for each intervention client based on this review. The plan of care can involve a number of actions including a clinic visit, telephone call with a health care provider, further assessment, sharing information about or a referral to community resources. The TAP-Huddle can deliver the plan of care themselves and/or request a volunteer to help (e.g., help clients sign up for a community program). The TAP-Huddle can share a patient-friendly report (in plain language) with the client containing the patient’s goals, open-ended responses, and next steps suggested by the TAP-Huddle. At the end of the 6 months, the Health TAPESTRY volunteers will complete another visit with clients to complete the surveys for a second time.

#### Control group

Once the baseline visit is complete, control clients will receive usual care. No report will go to the TAP-Huddle until the client received the intervention, nor will any volunteer follow-up take place. After, 6-month research outcomes are collected, and clients will be offered the Health TAPESTRY program.

#### Volunteers

To manage the incorporation of trained volunteers into the program across sites, we formed a partnership with the Canadian Red Cross and the Windsor Essex Compassion Care Community to recruit, train, manage, and retain the community volunteers. All volunteers will undergo a screening process. Volunteers meeting screening criteria will receive a blended-model training program of online and in-person training specific to the Health TAPESTRY program. A full description of the volunteer program has been published elsewhere [50].

### Strategies to improve adherence to protocol

The Health TAPESTRY research team will continually monitor study participant recruitment and timelines. Bi-weekly meetings with the study team and volunteer coordinators will be held to ensure fidelity to the trial protocol and huddle process. Volunteer coordinators will provide general oversight for the TAP-Reports and address any issues volunteers may experience related to the visits. Continuing education opportunities for volunteers will be provided to clarify procedures, refresh information from initial training, and provide new information as knowledge and self-efficacy gaps become apparent. Deviations from the protocol will be documented in meeting minutes.

### Data collection

Outcome data collection will occur through the structured surveys via the TAP-App, from the electronic medical record (EMR), program records, and focus groups/interviews (Table 3). Health care utilization outcomes will be measured during the 6-month period prior to study enrollment (i.e., before baseline) and during the 6-month study period. For the EMR data extraction, all researchers will undergo training and use a standardized data abstraction form that has been pilot tested. We will calculate agreement scores between auditors for a subset of the sample.

**Table 3:** Summary of outcomes, measures, and analysis plan using the RE-AIM framework

RE-AIM element	Outcome	Outcome measure; *source*	Data collection time point	Analysis
Reach *The reach of the intervention into the target population*	Participants	Proportion of eligible patients who consent; *self-report*	T_0_	Simple proportions and range across sites
	Sample characteristics	Demographics including chronic conditions; *self-report*, *EMR*	T_0_	Simple proportions and range across sites
	Volunteer visits	Number of volunteer visits; *program records*	T_6_	Frequency count across sites
Effectiveness *Positive and adverse effects of the intervention*	Hospitalizations*	Number of hospitalizations; *EMR*	T_0_, T_6_	Poisson regression or negative binomial regression
	Physical activity*	Total minutes spent doing moderate, vigorous, activity and walking (IPAQ); *self-report*;	T_0_, T_6_	Multiple linear regression
	Sitting	Hours sitting (IPAQ); *self-report*	T_0_, T_6_	
	Patient enablement	PEI; *self-report*	T_0_, T_6_	
	Quality of life	EQ 5D-5L; *self-report*	T_0_, T_6_	
	Treatment burden	MTBQ; *self-report*	T_0_, T_6_	
	Disease burden	DBMA; *self-report*	T_0_, T_6_	
	Emergency room or urgent care	Number of emergency room or urgent care visits; *EMR*	T_0_, T_6_	Poisson regression or negative binomial regression Poisson regression or negative binomial regression
	Falls	Number of falls; *EMR*	T_0_, T_6_	
	Medications	Number of medications; *EMR*	T_0_, T_6_	
	Primary care visits	Number of primary care visits; *EMR*	T_0_, T_6_	
	Negative effects	Unmet expectations; *self-report*	T_6_	Descriptive analysis across sites
		Labeling effect of screening tools; *self-report*	T_6_	
		Number and nature of serious adverse events; *EMR*	T_6_	
Adoption *Representation of settings and intervention agents who are willing to initiate and actively participate in program*		Number of health care providers who consent to participate; *program records*	T_6_	Simple proportions across sites
		Proportion of health care team members participating by health care profession; *program records*	T_6_	Simple proportions across sites, across professions
		NoMAD tool^£^ (NPT traffic light process); *program records*	T_1_, T_2_, T_3_, T_6_, T_9_, T_12_	Descriptive analysis across sites
		Number of volunteers recruited, trained, active, dropouts: *program records*	T_6_	Simple proportions across sites
Implementation *Fidelity to the intervention and adaptations*	Consistency of delivery as intended	Number of home visits, reports sent to clinic, number and nature of actions from TAP-Huddle; *EMR* Fidelity checklist; *program records*	T_6_	Frequencies and/or proportions across sites where appropriate
	Cost effectiveness	Program costs; *program records* QALYs; *self-report*	T_6_	Economic evaluation
	Barriers and facilitators or adaptations of implementation	*Focus groups/interviews, program records*	T_6_ – T_12_	Descriptive thematic analysis
Maintenance *Extent to which program becomes sustained over time*	Extent that program becomes institutionalized, part of practice or policies created	Proportion of patients and team members who recommend program;* self-report*	T_6_	Simple proportions across sites
		Indication of sites continuing program; *program records*	T_12_	Frequency count across sites
		NoMAD survey^£^; *self-report*	T_12_	Descriptive analysis across sites

*EMR* electronic medical record, *NPT* Normalization Process Theory, *QALY* quality-adjusted life year, *T*_*0*_ baseline, *T*_*6*_ 6-month data collection time point, *T*_*12*_ 12-month data collection time point

*Primary outcomes for the study

^£^Based on Normalization Process Theory

#### Study outcomes

We aim to determine the reproducibility of the effectiveness of the Health TAPESTRY program, as well as the implementation in six primary care practices. All study outcomes are mapped onto the RE-AIM framework, along with the data source (EMR, self-report, or program records) in Table 3 [30].

Reach: to determine the reach of the study into the target population. Relevant client characteristics will be assessed. Additionally, the proportions of eligible patients who participate and number of volunteer visits will be reported. This data will be collected from the TAP-App and program records.

Effectiveness: the impact of Health TAPESTRY on patient outcomes. The two primary outcomes, the number of hospitalizations during the 6-month study period and total physical activity per week (described below) at 6 months, were chosen based on results from the initial RCT [24]. Number of hospitalizations will be extracted from the EMR. The reason for each hospitalization at discharge will be categorized into ambulatory care sensitive conditions or acute care conditions as used to understand the nature of hospitalizations [51–53]. Physical activity will be measured using the short form version of the International Physical Activity Questionnaire (IPAQ), using the standard methods of calculating metabolic equivalent of task (MET) [38]. Secondary outcomes will include time sitting, patient enablement, quality of life, treatment burden, disease burden, emergency room/urgent care visits, falls, medications, primary care visits, and negative effects (see Table 3 for measures).

Adoption: the proportion of physicians and health care providers who consent to participate and a description of volunteer involvement throughout the study will be reported. In addition, to assess how Health TAPESTRY is taken up as normal practice in each site, the validated Normalization Measure Development (NoMAD) survey will be used [54]. TAP-Huddle members will answer questions related to implementation processes every 3 months for 1 year.

Implementation: three different ways to understand “implementation” will be completed. First, a fidelity checklist specific to this study will be used and completed during later implementation. The two-part checklist was developed by the research team based on reviewing the fidelity literature and a team discussion about the core program components. One part of the checklist specifically assesses the functioning of the TAP-Huddle. Items were generated from reviewing the literature on “best practices” for team-based care applicable to the TAP-Huddle (communication, roles, organizational support/resources, as well as processes deemed critical to Health TAPESTRY). The second part contains items related to the 4 core parts of Health TAPESTRY. All questions are scored as yes or no. The second way we will understand implementation is by interpreting the qualitative data. Primary health care team members will be invited to participate in focus groups/interview (stratified by site and role in Health TAPESTRY) at least 6 months post-implementation. The question guide will be grounded in NPT [26, 27] and center on implementation barriers and facilitators, interprofessional teamwork, collaboration, and system navigation. All focus groups/interviews will be audio-recorded and transcribed into intelligent verbatim. Finally, the third way to understand implementation is to determine the program’s value for money in regard to costs and quality-adjusted life years (QALYs).

Maintenance: we will report the proportion of providers and patients who would suggest the program to others, each implementation site’s interest in continuing the program after the study has ended. The NoMAD survey results will reflect the normalization of Health TAPESTRY into current practice by each provider and site. It will also be used as a reflection exercise for sites throughout the study by identifying areas for improvement based on collective survey responses.

### Sample size

We estimated the sample sizes for both co-primary outcomes physical activity and number of hospitalizations for different effect sizes, based on the initial trial [24, 25], using Bonferroni adjustment [55], with *α* = 0.025 and power = 0.80, using software PASS v19.0.4 [56]. We selected the total sample sizes 488 for physical activity (corresponding to mean difference = 403 and SD = 1441) and 426 for number of hospitalizations (corresponding to mean difference = − 0.14 and SD = 0.47). Using the larger sample size estimate for physical activity and accounting for 20% lost to follow-up, we will require a sample size of 586 participants.

### Data analysis

The results will be reported according to the CONSORT extension for pragmatic randomized trials [57] and non-pharmacological interventions [58]. The mean (standard deviation) or median (first quartile, third quartile) for continuous variables, depending on the distribution, and count (percent) for categorical variables will be calculated. The description of the data analysis approach below is separated based on the RE-AIM framework for effectiveness and implementation. Note that for reach, adoption, and maintenance, the analysis approach is found in Table 3.

Effectiveness: the effectiveness of Health TAPESTRY will be assessed at 6 months. We will adopt intention-to-treat (ITT) as the primary analysis approach. Multiple imputation approach using chained equations will be used to impute the missing data [59]. We will consider the fraction of missing information and determine the number of imputations needed using the two-stage approach suggested by Hippel [60]. The pooled effect estimates along with 95% confidence intervals will be reported. The co-primary outcome of a number of hospital admission will be analyzed using the Poisson regression or negative binomial regression depending on the distribution. The incidence rate ratio along with 95% confidence interval will be reported. And the co-primary outcome physical activity will be analyzed using the multiple linear regression. The mean difference between intervention and control group along with 95% confidence intervals will be reported. The continuous outcomes (sitting, patient enablement, quality of life, treatment and disease burden scores) and the count secondary outcomes (number of falls, number of primary care visits, number of emergency room or urgent care visits, number of medications) will be analyzed using the multiple linear and Poisson regression or negative binomial regression, respectively. The mean difference and incidence rate ratio along with 95% confidence intervals will be reported for continuous and count outcomes respectively.

All analyses will be adjusted for baseline values and sites. All statistical tests will be two-sided and all *p* values will be reported to three decimal places with those less than 0.001 reported as *p* < 0.001. The criterion for statistical significance will be set a priori at alpha = 0.05 and will be adjusted using the Bonferroni method for multiple testing for the co-primary outcomes. There will be no adjustment of alpha for secondary and subgroup analyses as these are exploratory. Analyses will be performed using R v3.6.1 [61].

We will perform sensitivity analyses of the primary outcomes to assess the effectiveness of Health TAPESTRY. We will adopt per-protocol approach to assess the effect of Health TAPESTRY at 6 months. In addition, we will assess the effect of Health TAPESTRY without adjusting for sites and baseline values. We will also use zero inflated Poisson or negative binomial distribution for the count data, depending on the distribution, to assess the robustness of the results.

Implementation: as noted above, implementation is assessed in three ways. For fidelity to the program, sites will be described as either high, medium, or low adherence to the program as intended. The qualitative data centered on implementation will be analyzed using a descriptive thematic analysis [62] and organized using NVivo 12 (QSR 2018) [63]. Transcripts will be independently coded inductively by two reviewers with qualitative analysis experience using open coding. A third reviewer will complete random code checking and provide oversight to ensure trustworthiness of the data. The interview questions will serve as the coding guide for the first few transcripts. The three reviewers will discuss and create a formal coding structure and review it regularly as more transcripts are coded to begin to collapse codes into over-arching themes. The themes will be aligned with the NPT constructs [26, 27].

To assess the third part of “implementation” of RE-AIM, an economic evaluation of the trial will be conducted in accordance with Canadian and international guidelines for the conduct of economic evaluations of healthcare programs [64–66]. This 6-month trial-based economic evaluation will compare Health TAPESTRY versus usual care in terms of costs and quality-adjusted life years (QALYs) from a public payer perspective. To calculate costs associated with the intervention, healthcare resource utilization (e.g., physician visits, emergency room visits, hospital visits) captured in the EMR (Table 3) will be multiplied by their respective unit costs. Cost associated with the delivery of Health TAPESTRY will be derived from trial data. The volunteer organizations will track front-line program costs (e.g., training, transportation costs for home visit) and research team will monitor all other costs (e.g., development of material, personnel time). Every site may not implement the program exactly the same in which case any cost differences will be accounted for. To measure the impact of Health TAPESTRY and usual care on health-related quality of life, all participants will answer the EQ-5D-5L at baseline and 6 months (Tables 2 and 3). The Canadian algorithm will be used to derive the EQ-5D health utility scores [67]. QALYs will be calculated by the weighted EQ-5D health utility scores by time spent in health state using an area under the curve approach.

Differences in costs and QALYs will be determined using parametric or non-parametric tests as appropriate, and bootstrap techniques will be used to deal with sampling uncertainty and generate 95% confidence intervals [68]. Cost-effectiveness acceptability curves will be used to present the probability of Health TAPESTRY to be cost-effective at different willingness-to-pay thresholds (e.g., $50,000/QALY gained; $100,000/QALY gained) [64, 66]. Several sensitivity analyses will be conducted to explore the impact of certain assumptions (e.g., cost of implementing Health TAPESTRY) on the results. Missing data will be imputed using multiple imputations [69]. The results will be reported as per the Consolidated Health Economic Evaluation Reporting Standards (CHEERS) guidelines [70].

#### Data management and confidentiality

All electronic information will be password protected and stored on password protected computers in secure networks or on the TAP-App or REDCap software [36]. A coding system will be used to protect identifiable information. Only the Health TAPESTRY research team and volunteer coordinators will have access to the master file containing the coding system. Any electronic transfer of data will be done using a secure HTTPS protocol to mitigate risks associated with transferring information over the internet.

#### Data monitoring, critical and adverse events

Any critical incidents that volunteers encounter during study visits will be reported and followed up by the appropriate personnel using a standard operating procedure. Critical incidents include situations that prevent the visit from proceeding such as injury to a volunteer or client and emergency situations. Critical incidents identified by volunteers during the study will be immediately reported and monitored until the issue has been resolved.

Program-related adverse events from any source will be recorded. Adverse events are defined as “Any event that requires in-patient hospitalization or prolongation of existing hospitalization, causes congenital malformation, results in persistent or significant disability or incapacity, is life-threatening or results in death” [71]. The research team will monitor data conduct and oversee data quality and provide independent outcome adjudication of adverse events as potentially related to intervention or not, and to provide an assessment of the safety data at 6 months before the intervention is provided to the wait list control group.

## Discussion

The proposed study aims to reproduce the findings of the first randomized controlled trial of Health TAPESTRY. We will also explicitly look at barriers and facilitators to implementation. By assessing Health TAPESTRY using the five domains of the RE-AIM framework, we aim to gain a comprehensive perspective on the potential spread and scalability of the program to the wider primary health care system.

This study’s co-primary outcomes, physical activity and hospitalizations, were selected based on the success of Health TAPESTRY on those outcomes in the initial evaluation trial. The way in which these outcomes are collected has limitations to acknowledge. First, physical activity will be captured using a self-report survey, on which patients typically over-estimate their physical activity levels [72]. However, the same survey will be used by both intervention and control groups and each time point. Additionally, many physical activity questionnaires have low to moderate correlations with physical activity monitors [73]. This study will use the short-form International Physical Activity Questionnaire, which has been found to be valid and reliable measure in multiple contexts and populations [38]. Second, hospitalizations and many of the secondary outcomes pertaining to the programs’ effectiveness will be extracted by auditing patient charts in the EMR. Chart audits are labor intensive, but give access to data not otherwise easily available [74, 75]. Since health care providers frequently over-report behaviors on self-report surveys [76], chart audits can provide more realistic patient data. Unfortunately, missing data in the chart will be a limitation which we will be unable to overcome.

We plan to carry out this study through FHTs, so if results are reproduced, implementation studies will be needed in other settings. Patients rostered to FHTs have access to diverse health care providers, whereas patients in other models of primary care practice may not have the same level of access. However, we anticipate the results will be generalizable to other FHTs in the Ontario health care system—approximately one quarter of attached patients in Ontario are rostered to a FHT [77]—and potentially generalizable to other primary care organizations that include team-based interprofessional care and other funding models. The FHTs participating in the study are from communities across Ontario, including both urban and rural locations, and they offer a variety of clinical programs and interprofessional to patients, creating a diverse sample of sites.

A strength of this evaluation is the inclusion of outcomes to assess possible negative outcomes associated with Health TAPESTRY, including disease burden, treatment burden, and the labeling effects of screening tools. Understanding not only the benefits of Health TAPESTRY but also the risks is important prior to spread or scale. This evaluation also includes several outcomes from the first trial so as to help to elucidate findings that were close to significant, such as falls. Another strength of the proposed study is that it will test reproducibility of results from a single site RCT in multiple diverse sites, as well as evaluate implementation. Many health care innovations are developed and found to be effective, yet are not sustained as part of routine care. This may be explained by a lack of evaluating the innovations described. Another strength is the evaluation of contributing factors to implementation (barriers and facilitators) that can be compared across sites. This will provide further insight into strategies to enhance implementation in the future and the potential need for adaptations of the intervention to address contextual factors (e.g., rural/urban, primary care team make up, local volunteer capacity). Our evaluation plan that addresses both effectiveness and implementation lessons, as well as other components of RE-AIM, will provide a solid foundation to guide the scaling of Health TAPESTRY to other communities and primary health care contexts in the future.

### Trial status and dissemination policy

This trial is in the recruitment phase, and we expect the final 6-month follow-up visit for the intervention and control participants to occur in mid-2020. Recruitment began on March 15, 2018, and is expected to conclude at the end of January 2020. The study is using protocol version 2, dated July 2018. The results of this study will be published in peer-reviewed academic journals and presented at academic conferences. The datasets analyzed during the current study will be available from the corresponding author on reasonable request.

## Supplementary information

**Additional file 1.** SPIRIT 2013 Checklist.

**Additional file 2.** TIDieR Checklist.

## Data Availability

Not applicable

## Abbreviations

CHEERS: Consolidated Health Economic Evaluation Reporting Standards
DBMA: Disease Burden Morbidity Assessment
EMR: Electronic medical record
FHT: Family Health Team
Health TAPESTRY: Health Teams Advancing Patient Experience: Strengthening Quality
IPAQ: International Physical Activity Questionnaire
ITT: Intention-to-treat
MET: Metabolic equivalent of task
MTBQ: Multimorbidity Treatment Burden Questionnaire
NoMAD: Normalization Measure Development
NPT: Normalization Process Theory
PEI: Patient Enablement Instrument
RAPA: Rapid Assessment of Physical Activity
RCT: Randomized controlled trial
TAP-App: Health TAPESTRY web-based application
TAP-Report: Health TAPESTRY report
RE-AIM: Reach, Effectiveness or Efficacy, Adoption, Implementation, Maintenance
