# Supplementary material for: Health TAPESTRY Ontario: protocol for a randomized controlled trial to test reproducibility and implementation
Source: Trials. 2020 Aug 14;21:714. doi: 10.1186/s13063-020-04600-y (PMC7427958; doi:10.1186/s13063-020-04600-y)
Supplement: Supplementary file 2 — Additional file 2. TIDieR Checklist. [file 13063_2020_4600_MOESM2_ESM.pdf]

Additional File 2. The TIDieR (Template for Intervention Description and Replication) Checklist

Item Number	Item	Where located<br>Primary paper (page or appendix)	Other† (details)
**Brief Name**			
1	Provide the name of a phrase that describes the intervention.	3	
**Why**			
2	Describe any rationale, theory, or goal of the elements essential to the intervention.	6,7	
**What**			
3	Materials: Describe any physical or informational materials used in the intervention, including those provided to participants or used in intervention delivery or in training of intervention providers. Provide information on where the materials can be accessed (e.g. online appendix, URL).	13-16, Table 2	
4	Procedures: Describe each of the procedures, activities, and/or processes used in the intervention, including any enabling or support activities.	11-12	The TAP-App was designed prior to this study, but will undergo modifications for this study.
**Who Provided**			
5	For each category of intervention provider (e.g., psychologist, nursing assistant), describe their expertise, background and any specific training given.	11	The participating interprofessional health care are existing employees of the implementation sites. The initial huddle members are trained by the research team and subsequent members are orientated by existing members.
**How**			

6	Describe the modes of delivery (e.g., face-to-face or by some other mechanism, such as internet or telephone) of the intervention and whether it was provided individually or in a group.	11-12	
**Where**			
7	Describe the type(s) of location(s) where the intervention occurred, including any necessary infrastructure or relevant features.	Table 1, 9	
**When and How Much**			
8	Describe the number of times the intervention was delivered and over what period of time including the number of sessions, their schedule, and their duration, intensity or dose.	11-12, Table 2	Sessions will be scheduled for one hour, but volunteers may return to a client's home if more time is needed.
**Tailoring**			
9	If the intervention was planned to be personalised, titrated or adapted, then describe what, why, when and how.	11-12	Each implementation site can select additional questionnaires they want to ask participants.
**Modifications**			
10‡	If the intervention was modified during the course of the study, describe the changes (what, why, when and how).	N/A	
**How Well**			
11	Planned: If intervention adherence or fidelity was assessed, describe how and by whom, and if any strategies were used to maintain or improve fidelity, describe them.	13, 15	
12‡	Actual: If intervention adherence or fidelity was assessed, describe the extent to which the intervention was delivered as planned.	N/A	

Note. N/A = an item is not applicable for the intervention being described; EMR = electronic medical record; PHR = personal health record; TAP-App = Health TAPESTRY application used during home visits; VLC = virtual learning centre used for volunteer training.

† The information is not provided in the primary paper, instead is provided in the table.

‡ If completing the TIDieR checklist for a protocol, these items are not relevant to the protocol and cannot be described until the study is complete.
